# Differences in Eysenck’s Personality Dimensions between a Group of Breast Cancer Survivors and the General Population

**DOI:** 10.3390/ijerph16071240

**Published:** 2019-04-08

**Authors:** Francisco García-Torres, Rosario Castillo-Mayén

**Affiliations:** 1 Department of Psychology, Faculty of Educational Sciences, University of Cordoba, 14071 Cordoba, Spain; z92camam@uco.es; 2 Maimonides Biomedical Research Institute of Cordoba, IMIBIC, 14004 Cordoba, Spain; 3 Reina Sofía University Hospital, 14004 Cordoba, Spain

**Keywords:** oncology, breast cancer, survivorship, personality, psychoticism

## Abstract

Cancer may influence personality in patients and survivors. However, the possible relations between the treatments that the patients have undergone and the personality in survivors are not clear. This study aimed to establish the differences in personality between a group of breast cancer survivors and a control group, and to test the predictive utility of the treatments on the personality traits in survivors. Thirty breast cancer survivors and thirty participants from the general population completed the Eysenck personality questionnaire-revised (EPQ-R) and a socio-demographic questionnaire. Survivors had lower scores on extraversion and higher scores on neuroticism than the control group, but these differences were not significant. However, differences in psychoticism were significant, with higher scores in the survivor group. Breast-conserving therapy predicted extraversion while breast reconstruction predicted psychoticism. These results suggest that the physical consequences of surgery may lead to social and psychological impairments in this group of patients.

## 1. Introduction

Personality is described as a way of perceiving, relating, and thinking about the environment and oneself. The concept of personality includes different traits, which can be described as relevant aspects of personality that are present in a wide range of situations and contexts, and these traits are relatively stable over time [1,2], but may be influenced by traumatic events such as cancer [3]. According to Eysenck’s theory, personality includes three major dimensions that are related with biological systems. Extraversion is related with differences in the atablescending activating reticular system, with low activity for extraverts and high levels for introverts. Neuroticism is also related with differences in limbic system reactions of people, with high levels of neuroticism in people with high sensitivity in that system. Finally, the explanation about psychoticism is less clear. It looks as though dopamine levels are higher in people with high psychoticism, but the evidence to support this affirmation is still under discussion [4]. Previous research has applied Eysenck’s personality questionnaire in cancer survivors, showing that neuroticism and psychoticism are related to relevant aspects of survival, such as fatigue, poor quality of life, and depression [5,6,7,8]. A longitudinal study using the EPQ questionnaire showed that neuroticism was negatively related to survival rates through a possible dysregulation of the immune system [9]. Only a few studies have evaluated whether personality in breast cancer survivors is different to personality observed in the general population, probably due to methodological problems and the supposed stability of these dimensions. 

In this line of research, previous data suggests that cancer patients have higher levels of neuroticism and psychoticism when compared with the general population one year after completion of treatment, probably due to work changes and high distress levels [10,11]. As for breast cancer survivors, research has shown that patients who had mostly undergone surgery, including mastectomy, showed significantly lower levels of extraversion and higher levels of psychoticism, and these differences have been explained by the fact that psychological distress is highly likely to be a consequence of the surgery [8,12,13], suggesting that the type of treatment may influence these personality dimensions in survivors. However, little data is available to support this affirmation at this moment. Accordingly, given that it is important to know the potential psychological consequences of oncologic treatments, this research has the following objectives: firstly, to determine whether the presence of a traumatic event like breast cancer affects personality dimensions (extraversion, neuroticism, and psychoticism) in survivors, and, secondly, to examine whether the type of medical treatment (mastectomy, chemotherapy, radiotherapy, breast-conserving therapy, and breast reconstruction after surgery) that patients underwent has a predictive value on personality variables. Thus, the hypotheses of the study were as follows: (1) cancer survivors will show higher levels of neuroticism and psychoticism and lower levels of extraversion than the general population, and (2) medical treatments, especially those that involve surgery, will be able to partially explain personality dimension levels in breast cancer survivors.

## 2. Materials and Methods

### 2.1. Participants

The participants of this study consisted of sixty-two women who were divided into two groups. Thirty-two breast cancer survivors were invited to join the study based on the condition that they had completed their treatment. Most of the patients had received more than one treatment: mastectomy (80%), chemotherapy (73%), radiotherapy (43%), hormonal therapy (33%), breast-conserving therapy (16%), and breast reconstruction (15%). The time from the end of treatment was between one and 21 years, with an average of 8.90 years (SD = 5.89). All patients were in stages I–III and free of metastases or relapse. The average age was 58.70 years (SD = 7.77). Ultimately, thirty breast cancer survivors agreed to participate in the study. For the control group, thirty women were chosen who had not experienced any type of cancer; their average age was 54.96 years (SD = 11.92).

### 2.2. Instruments

The participants completed the full Spanish version of the Eysenck Personality Questionnaire-Revised (EPQ-R) [4]. This questionnaire assesses three features of Eysenck’s theory of personality, and includes an additional scale that measures the sincerity/compliance of responses. It is an 83-item self-administered questionnaire with a yes/no format. Once all responses are combined, raw scores of each of the scales are transformed into *T* scores, from which the results are interpreted. *T* scores are classified as follows: 1–35 Very Low, 36–45 Low, 46–55 Average, 56–65 High, and 66–99 Very High. High scores indicate a tendency to possess the personality trait represented by each subscale. Extraversion represents sociability and liveliness; neuroticism represents emotional instability and anxiety; psychoticism indicates aggressiveness, coldness, and egocentricity, and sincerity/compliance is represented by acceptance of social norms. The reliability of the different scales is adequate in the Spanish population: Extraversion (α = 0.80), Neuroticism (α = 0.86), Psychoticism (α = 0.71), Sincerity/Compliance (α = 0.77) [4,14]. Furthermore, the participants carried out a socio-demographic questionnaire that retrieved information about age, civil status, employment status, education, treatment, and time from the end of the treatment.

### 2.3. Procedure

We contacted the association against cancer in Cordoba (Spain) asking for permission to conduct the study. After receiving the ethical committee’s approval, professionals of the association were asked to provide participants meeting the criteria. When interested participants were identified, the association provided our contact number so that they could make an appointment. Thirty-two patients were invited to participate. Two declined the invitation; the reason cited in both cases was: “I don’t want to remember the cancer”. Thirty volunteers from the same association were invited to participate as controls and all of them agreed to participate. Both groups were provided with a written informed consent form which provided information regarding the objectives of the study and outlined the confidentiality of the results. 

All subjects gave their informed consent for inclusion before they participated in the study. The study was conducted in accordance with the Declaration of Helsinki, and the protocol was approved by the Ethics Committee of the Spanish Association Against Cancer.

### 2.4. Statistical Analysis

To test the differences between the groups in sociodemographic variables, we applied *χ*^2^ and independent samples *t*-test. Furthermore, we tried to find differences between the groups in personality scores using *t*-test. Finally, a hierarchical multiple regression analysis was performed for neuroticism and extraversion dimensions, with age in the first block using forced entry and the treatments in the second block using a stepwise method, as these personality dimensions are sensitive to change over time [15]. Concerning psychoticism, only the treatments were used as predictors. Results were considered significant with *p* < 0.05.

## 3. Results

First, we analyzed the equivalence of the two groups in the socio-demographic variables examined. The two groups differed only in employment status; most of the participants in the survivors group were retired and the majority of the participants in the control group were unemployed (*χ*^2^ (2, *N* = 60) = 10.72, *p* = 0.005). Extraversion and neuroticism scores were at average levels in both groups; survivors showed lower levels of extraversion and higher levels of neuroticism but with no significant differences. Psychoticism and sincerity/compliance were in the high category in the survivors group, and sincerity/compliance was in the high category in the control group. For the remaining dimensions, all scores were in the average range in both groups. Statistical differences were only observed in psychoticism (see Table 1).

In addition, we evaluated the predictive ability of the treatment to which the patients were subjected (mastectomy, chemotherapy, radiotherapy, breast-conserving therapy, and breast reconstruction) on the personality dimensions assessed. Our results showed that, after controlling for age (*R*^2^ = 0.003), breast-conserving therapy explains more than 30% of the variance on extraversion (Δ*R*^2^ = 0.312). Additionally, breast reconstruction explained 29% of the variance of psychoticism scores (see Table 2). Concerning neuroticism, any of the models showed a significant contribution, *p* > 0.05.

Finally, in the socio-demographic variables, only age was correlated with sincerity/compliance in survivors (*r*(30) = 0.57, *p* < 0.001).

## 4. Discussion

Personality in cancer survivors has been related to key aspects of survival such as depression, quality of life, and fatigue [5,6,7,8]. However, there is a paucity of information about the influence of cancer on the relative stability of the personality dimensions of survivors. For this reason, we tried to find differences in three personality dimensions between a group of breast cancer survivors and the general population using Eysenck’s personality test. Thus, regarding our first hypothesis, we observed a similar pattern to those previously identified in breast cancer survivors [8,12,13], with lower scores in extraversion and higher scores in neuroticism and psychoticism when compared with a control group, and statistical differences in psychoticism. To explain these results, we tried to establish the predictive ability of the different treatments on personality scores. Accordingly, in relation to our second hypothesis, the results showed that breast-conserving surgery predicted extraversion, after controlling for age, and breast reconstruction predicted psychoticism. It is possible that the physical consequences of cancer in patients who had undergone those procedures may lead the patients to some kind of social retrieval, as extraversion and psychoticism include traits related to social function (i.e., sociability, coldness, egocentrism) because patients may feel that they are different from their peers as a consequence of cancer [16]. Interestingly enough, a high percentage of the patients in the study sample had undergone mastectomy, however, there is no predictive utility of this treatment on any personality dimension. It is possible that in patients who had undergone mastectomy the effects of this treatment are more related to psychological disturbances such as depression than to personality issues [17]. Overall these results confirm the relevance of taking into account the type of treatment that patients receive and the necessity of fostering communication skills as preventive strategies in patients and strengthening their social bonds in order to reduce the social impairments that may appear in these patients. The dimension of sincerity/compliance reached high values in both groups, but when interpreting scores on this scale, age in adults causes an increase in sincerity/compliance, which can be interpreted as a greater acceptance of social norms [4]. In both groups, the average age was over 50 years, so for this reason high scores on sincerity/compliance can be explained by age. To support this claim, we found significant and positive correlations between age and the level of sincerity/compliance in our study. 

## 5. Limitations of the Study

There were several limitations of this research. Firstly, some patients received more than one type of medical treatment and there may have been an interaction effect between them, which might hamper the interpretation of the results. Future research should consider this limitation and try to obtain a sample of participants who have been subjected to a single treatment condition. Secondly, the sample size needs to be expanded to ensure the generalization of results in this group of patients. Finally, the results obtained must be confirmed using longitudinal studies and with a baseline evaluation of personality scores in patients.

## 6. Conclusions

Our study, according to previous data, confirms that the differences between personality traits of survivors and the general population are significant. However, the way in which cancer diagnosis and treatment can affect personality traits remains unclear. Further research explaining these problems are needed. These results can be helpful in preventing personality and behavioral changes in cancer survivors by helping them to implement preventive psychosocial strategies.

## Figures and Tables

**Table 1:** Comparison of personality variables between groups.

Group	Extraversion M (SD)	Neuroticism M (SD)	Psychoticism M (SD)	Sincerity/Compliance M (SD)
Survivors	49.23 (9.35)	46.73 (9.57)	55.66 (8.58)	61.46 (9.16)
Control	50.46 (9.04)	43.36 (10.68)	50.46 (7.13)	60.86 (9.62)
	*t*(58) = −0.51, *p* = 0.60, *d* = 0.13	*t*(58) = 1.28, *p* = 0.20, *d* = 0.33	*t*(58) = 2.55, *p* = 0.01, *d* = 0.66	*t*(58) = 0.24, *p* = 0.80, *d* = 0.06

M: mean; SD: Standard Deviation. Differences are significant in psychoticism (*p* < 0.05).

**Table 2:** Regression analysis results for extraversion and psychoticism dimensions.

Model	*b*	SE *b*	*β*	*t*
*Extraversion* Model 1				
Constant	52.94	13.44		3.93 ***
Age	−0.06	0.23	−0.05	−0.28
Model 2 Constant	46.06	11.51		4.00 ***
Age	0.09	0.19	0.07	0.47
Breast-conserving therapy	−14.16	4.03	−0.57	−3.50 **
*Psychoticism* Constant	58.73	4.79		12.25 ***
Breast Reconstruction	8.63	4.06	0.47	2.26 *

Extraversion: *R*^2^ = 0.003 for Step 1; Δ*R*^2^ = 0.312 for Step 2 (*p* < 0.01). Psychoticism: *R*^2^ = 0.291. * *p* < 0.05, ** *p* < 0.01, *** *p* < 0.001.
